# Delayed Exercise Training Improves Obesity-Induced Chronic Kidney Disease by Activating AMPK Pathway in High-Fat Diet-Fed Mice

**DOI:** 10.3390/ijms22010350

**Published:** 2020-12-31

**Authors:** Florian Juszczak, Maud Vlassembrouck, Olivia Botton, Thomas Zwakhals, Morgane Decarnoncle, Alexandra Tassin, Nathalie Caron, Anne-Emilie Declèves

**Affiliations:** 1 Laboratory of Metabolic and Molecular Biochemistry, Faculty of Medicine and Pharmacy, Research Institute for Health Sciences and Technology, University of Mons (UMONS), 7000 Mons, Belgium; maudvlassembrouck@gmail.com (M.V.); Thomas.ZWAKHALS@umons.ac.be (T.Z.); Morgane.decarnoncle@student.umons.ac.be (M.D.); anne-emilie.decleves@umons.ac.be (A.-E.D.); 2 Molecular Physiology Research Unit (URPhyM), Namur Research Institute for Life Sciences (NARILIS), University of Namur (UNamur), 5000 Namur, Belgium; olivia.botton@unamur.be (O.B.); nathalie.caron@unamur.be (N.C.); 3 Laboratory of Respiratory Physiology, Pathophysiology and Rehabilitation, Faculty of Medicine and Pharmacy, Research Institute for Health Sciences and Technology, University of Mons (UMONS), 7000 Mons, Belgium; Alexandra.TASSIN@umons.ac.be

**Keywords:** high-fat diet, chronic kidney disease, endurance exercise training, AMPK, autophagy, ectopic lipid accumulation

## Abstract

Exercise training is now recognized as an interesting therapeutic strategy in managing obesity and its related disorders. However, there is still a lack of knowledge about its impact on obesity-induced chronic kidney disease (CKD). Here, we investigated the effects of a delayed protocol of endurance exercise training (EET) as well as the underlying mechanism in obese mice presenting CKD. Mice fed a high-fat diet (HFD) or a low-fat diet (LFD) for 12 weeks were subsequently submitted to an 8-weeks EET protocol. Delayed treatment with EET in obese mice prevented body weight gain associated with a reduced calorie intake. EET intervention counteracted obesity-related disorders including glucose intolerance, insulin resistance, dyslipidaemia and hepatic steatosis. Moreover, our data demonstrated for the first time the beneficial effects of EET on obesity-induced CKD as evidenced by an improvement of obesity-related glomerulopathy, tubulo-interstitial fibrosis, inflammation and oxidative stress. EET also prevented renal lipid depositions in the proximal tubule. These results were associated with an improvement of the AMPK pathway by EET in renal tissue. AMPK-mediated phosphorylation of ACC and ULK-1 were particularly enhanced leading to increased fatty acid oxidation and autophagy improvement with EET in obese mice.

## 1. Introduction

In the general population, obesity is the second most highly predictive factor for end-stage renal disease, independently of diabetes and hypertension [1]. Central obesity is related to caloric excess promoting deleterious cellular responses in targeted organs. Obesity is mainly caused by a sedentary lifestyle commonly characterized by an excess consumption of ultraprocessed food containing high amounts of saturated fat, and a lack of physical activity [2]. The prevalence of obesity is increasing worldwide and predictive projections suggest that nearly 50% of adults will be obese by 2030 [3,4]. The consequences of adiposity include the endocrine activity of the adipose tissue via the production of adipokines, leading to the development of inflammation, oxidative stress, activation of the renin-angiotensin-aldosterone system and increased insulin resistance in the kidney [5]. Clinical and experimental studies have also demonstrated obesity-related glomerulopathy, which is characterized by glomerulomegaly with thickening of the glomerular basement membrane, mesangial matrix expansion, hyperfiltration-related glomerular filtration barrier injury and, ultimately, focal glomerulosclerosis [6,7,8]. Furthermore, studies showing ectopic lipid depositions in the kidney have emerged, suggesting a role of fat accumulation in the development and progression of CKD [9,10,11,12]. Direct intrarenal effects of obesity involve lipid accumulation and impaired fatty acid β-oxidation (FAO) in kidney cells and tissue [13]. Renal proximal tubular cells (PTC) are metabolically very active, requiring a large amount of ATP, which is mostly provided through the mitochondrial FAO. In a previous work, we demonstrated a robust accumulation of lipids within enlarged multilamellar inclusions (MLI) into the PTC along with impaired tubular function and increased oxidative stress in obese mice [10]. Yamamoto et al. also described the stagnation of the autophagy flux in PTC, associated with the impairment of the lysosomal system. These alterations likely contribute to albuminuria, a progressive decline in renal function and end-stage renal disease. Moreover, evidence of a dysregulation of AMPK activity in podocytes and proximal tubular cells in diabetic and obesity conditions has been demonstrated [14,15]. In addition, pharmacological AMPK activation has been shown to improve pathologic features of the disease [10]. AMPK is a ubiquitous heterotrimeric enzyme that is the master energy sensor in all eukaryotic cells and is abundantly expressed in kidneys [16]. It is a central mediator of energy homeostasis responsive to nutritional and metabolic stresses such as obesity [17]. AMPK also activates autophagy through phosphorylation of ULK-1 in renal cells [18,19]. Therefore, inhibition of AMPK in kidney tissue is associated with poor outcomes.

Despite considerable efforts in the development of new therapeutic strategies, there is still a lack of effective treatment without strong side effects. Current pharmacotherapies that target food intake regulation have side effects (psychiatric and/or cardiovascular side effects) observed for long periods of use and may be associated with weight regain once the medication is stopped [20]. In addition, even though AMPK has been demonstrated to represent a key target for the treatment of metabolic diseases, indirect AMPK activators such as AICAR (5-Aminoimidazole-4-carboxamide ribonucleotide) have been widely investigated during the last decade by our group and others but did not demonstrate adequate and efficient therapeutic effects in clinical use [21]. The ongoing development of direct and specific AMPK activators thus represents an important pharmacological challenge [22]. This highlights the urgent need for alternative therapeutic strategies. Recently, the study of the effects of behavioral interventions such as exercise training in obesity-related diseases has regained interest. Indeed, targeting key pathways that could mediate beneficial effects by exercise represents a safer alternative therapeutic approach for the treatment of chronic metabolic disorders. Exercise training has been shown to exert beneficial outcomes in managing obesity-associated diseases [23,24]. However, there is still a lack of knowledge about the underlying mechanisms. Moreover, the efficacy of exercise-based therapeutic approach in patients presenting CKD remains controversial, mainly due to the experiment biases of the clinical studies regarding exercise training in obese patients with CKD [25,26,27,28].

Here, we evaluated for the first time, the potential therapeutic effect of a delayed exercise-based therapy on a treadmill in an obesity-induced CKD mice model. We further investigated the regulation of the AMPK pathway in response to the treatment.

## 2. Materials and Methods

### 2.1. Animals and Diet

The study was conformed to APS’s guiding principles in the Care and Use of Animals and was approved by the Animal Ethics Committee of the University of Mons (DE-01-01; 09-01-2017). Experiments were performed on eight weeks old C57Bl/6J male mice (Janvier Labs, Le Genest Saint-Isle, France) housed in cages with ad libitum access to water and food and were maintained at 35–40% relative humidity and a temperature of 20–23 °C in a 12:12 h light–dark cycle. Eight-week old male mice were randomized either to a low-fat diet (LFD–10% of total calories from fat; D12450J, Research Diets, New Brunswick, NJ, USA) or a high-fat diet (HFD–60% of total calories from fat; D12492, Research Diets, New Brunswick, NJ, USA). Food intake and body weight were measured weekly during a 20 weeks exposure period. At week 13, LFD and HFD mice were randomized in either of two additional groups submitted to an endurance exercise training (EET) for eight weeks (LFDT and HFDT) or the untrained control groups (LFD and HFD) (*n* = 10 in each group). Mice fed a high-fat diet for 12 weeks were previously described to present obesity-related metabolic disturbance as well as characteristic features of obesity-induced CKD [10], and were used to evaluate exercise training protocol as a therapeutic strategy.

### 2.2. Exercise Training Protocol

After 12 weeks of the experimental protocol, trained groups (LFDT and HFDT) were exercised for a total of eight weeks (five days a week) as previously described in [29]. Briefly, mice were acclimated to a treadmill (Treadmill Control LE8700, Panlab apparatus^®^, Barcelona, Spain) at a speed of 3 m/min for 5 min and 9 m/min for 10 min during weeks 13 and 14. At the beginning of week 15, a maximal running velocity test was performed for each trained mouse with a gradual speed increase of 1.2 m/min every two min until exhaustion (defined as a maximum of four electric stimulations in one minute). Then, the running velocity (Vmax) was set at 70% of the maximal speed for each mouse. As shown in Supplementary Materials, the maximal running velocity (Vmax) was similar in both trained groups of mice, LFDT and HFDT respectively, indicating that ET had no impact on exercise performance in obese mice (Figure S1). Therefore, mice from LFDT and HFDT were trained with an equivalent running velocity (70% of the Vmax) throughout the EET protocol. The exercise duration started at 10 min/day and was increased by 10 min every week. Sedentary mice were not exposed to exercise training and stayed in their cages during exercise sessions.

### 2.3. Sample Collection

Mice were euthanized after 20 weeks on the diet. Blood samples were collected by intracardiac puncture and centrifuged at high speed for 20 min at 4 °C. Plasma was collected and stored at −80 °C until use. Liver, heart and kidney were collected, weighed and immediately processed for further analysis. Portions of tissues were snap-frozen in liquid nitrogen for RNA, protein extraction and lipid content quantification. Additional portions of tissues were fixed in Duboscq-Brazil solution for histological analysis.

### 2.4. Urine Collection and Measurement of Urinary Markers

At weeks 0, 12 and 20 of the protocol, mice were placed into metabolic cages for 24 h-urine collection. A mouse Albuwell ELISA kit and a Creatinine Companion kit (Exocell, Philadelphia, PA, USA) were used to determine the urinary albumin to creatinine level for each mouse at the different timepoints. Total proteinuria was quantified using the Bradford binding assay, as previously described [30]. As an index of oxidative stress, urine samples were also analyzed for hydrogen peroxide by Amplex red assay (Thermo Fisher Scientific, Waltham, MA, USA) and for 8-hydroxy-2-deoxy guanosine (8-OH-dG) by competitive ELISA (Gentaur, Kampenhout, Belgium) following the manufacturer’s instructions, and the data were normalized to the urinary creatinine from each mouse.

### 2.5. Glucose Tolerance Test

After a 12 h-overnight fast and 18 h after the last exercise session, a glucose tolerance test (GTT) was performed at weeks 0, 12 and 20 of the experimental protocol. A dose of 2 g/kg body weight of d-glucose (Roth, Karlsruhe, Germany) was administered intraperitoneally. Blood samples were then obtained from the caudal vein, and the blood glucose level was measured at 0, 30, 60, and 120 min after glucose injection using a One Touch^®^ Verio^®^ glucometer (Zug, Switzerland).

### 2.6. Biochemical Assays

ELISA. Plasma insulin level was determined by ELISA with the Rat/Mouse Insulin ELISA kit (Merck, Darmstadt, Germany). The homeostasis model assessment (HOMA) for insulin resistance index was determined using a calculator available from the Oxford Centre for Diabetes, Endocrinology and Metabolism (https://www.dtu.ox.ac.uk/homacalculator/). Plasma Adiponectin concentration was measured according to the manufacturer’s instructions (Adiponectin: MRP300, R&D Systems, Minneapolis, MN, USA). Plasma TNFα concentration was measured according to the manufacturer’s instructions (TNF alpha (mouse) ELISA kit; Gentaur, Kampenhout, Belgium).

Plasma lipid levels. Colorimetric enzymatic tests were performed to measure plasma triglycerides (TG) and cholesterol levels (Diasys, Diagnostic System, Holzheim, Germany) and plasma nonesterified fatty acids (NEFA) level (Wako Pure Chemical Industries, Ltd., Osaka, Japan) following the manufacturer’s instructions.

Kidney and liver lipid levels. 90 mg of frozen kidney or liver tissue were prepared as described in [10]. Briefly, renal tissues were homogenized in a dounce homogenizer (Tenbroeck, Kimble/Kontes Glass Co., Vineland, NJ, USA) at 4 °C with nitrogen-sparged acid methanol: [0.2 N HCl/0.2 M NaCl] (4:1 mix) solution. For the first extraction phase, chloroform was added followed by a 30 s vortex to denature and extract proteins. The second extraction phase was performed by adding chloroform/water (2.5:3) to the mix. The samples were centrifuged at 16,000 *g* for 10 min at 4 °C to separate the phases. The lower phase (chloroform) containing lipids was used for measurement of total triglycerides and cholesterol (Diasys, Diagnostic System, Holzheim, Germany) according to the manufacturer’s instructions. NEFA level was measured in the same phase using a colorimetric enzymatic test according to the manufacturer’s instructions (Wako Pure Chemical Industries, Ltd., Osaka, Japan).

### 2.7. Morphological Analysis

Paraffin-embedded kidney sections were stained with Periodic Acid Schiff (PAS), Hemalun, and Luxol fast blue for morphological analysis. Morphometry of kidney sections was carried out as previously reported [15]. The glomerular area, mesangial matrix expansion and nuclei count were determined by randomly analyzing 25 glomeruli in the outer cortex in each kidney section using ImageJ based on [31]. In addition, a semiquantitative single-blind analysis was performed to evaluate the frequency of vacuolated tubular cells in renal cortex using an additional lens engrave with a square grid inserted in one of microscope eyepieces. For each paraffin section, 10 square fields (0.084 mm^2^/field) were analyzed at ×400 magnification. In order to evaluate tubule-interstitial fibrosis, paraffin-embedded kidney sections were stained with picrosirius red (collagen I and III deposits). Ten randomly selected area of each kidney section were then analyzed with imageJ to measure the percentage of positive area. Paraffin-embedded liver sections were stained with hematoxylin and eosin and steatosis was graded as described by Ryu et al., 2015 [32].

### 2.8. Immunohistochemistry

Paraffin-embedded kidney sections were dewaxed and rehydrated followed by a microwave pretreatment in 1 mM EDTA buffer to unmask antigens present in the renal tissue. Endogenous peroxidase activity was removed by incubation with 3% H_2_O_2_ for 10 min and blockaded with 10% normal goat serum. Sections were incubated with primary antibody against LAMP-1 (Abcam) overnight at 4 °C. After rinsing in TBS, slides were exposed for 30 min with SignalStain^®^ Boost IHC Detection Reagent (Cell Signaling, Danvers, MA, USA) and bound peroxidase activity was detected with the DAB kit (Agilent DAKO, Heverlee, Belgium). Counterstaining was performed with hemalun and Luxol fast blue. For each section, 10 square fields (0.084 mm^2^/field) were observed at ×400 magnification in the renal cortex. The randomly selected area of each kidney section were then analyzed with imageJ software. The relative area occupied by positive staining was expressed as a percentage.

### 2.9. Quantitative Real-Time PCR

Snap-frozen kidney tissues were homogenized, and total RNA was then extracted with Trizol (Sigma-Aldrich, Saint-Louis, MO, USA) and treated with DNAse (Promega, Madison, WI, USA). Then, total RNA concentration was measured using NanoDrop (NanoDrop 1000, Thermo Scientific, Waltham, MA, USA) and 2 μg of total RNA were used for reverse transcription using MLV reverse transcriptase (Promega) following the manufacturer’s instructions. Real-time quantitative PCR was performed in order to quantify mRNA level of *Col I*, *Col III*, *TGFβ*, *MCP-1*, *IL-1β*, *TNFα*, *IL-6*, *ACC*, *CPT-1*, *FAS* and *18S* as a housekeeping gene (see Table S1). Quantitative PCR amplification of a triplicate for each gene was performed using the SYBR Green Master Mix (Roche, Basel, Switzerland). Relative gene expressions were calculated using the 2^−ΔΔCT^ method.

### 2.10. Western Blot Analysis

Frozen kidney tissues were homogenized in Cell Lysis Buffer (Cell Signaling, Danvers, MA, USA) with phosphatase and protease inhibitor cocktail (Thermo Fisher Scientific, Waltham, MA, USA) at 4 °C. Samples were subsequently centrifuged at 14,000× *g* for 15 min at 4 °C and supernatants were collected. Protein concentrations were quantified by Pierce BCA assay kit (Thermo Fisher). Next, 40 µg of total lysate were separated on Tris-Glycine 12% or Tris-acetate 3–8% (for high-molecular weight proteins) gels and transferred onto nitrocellulose membranes. The relative amounts of LMW, MMW and HMW Admer in plasma were determined using a nondenaturing PAGE-SDS as described in [29]. Membranes were blocked with 5% BSA for 1 h and primary antibodies against: phospho-ACC and ACC (Cell Signaling), phosphor-LKB1 and LKBA (Cell Signaling) phospho-AMPK and AMPK (Cell Signaling), CPT-1 (Abcam), Adiponectin (Abcam), Beclin-1 (Cell Signaling), p62 (Cell Signaling), phospho-ULK1 (Ser555) and ULK1 (Cell Signaling) and β-actin (Thermo Fisher) were applied in 5% BSA overnight at 4 °C. Finally, membranes were incubated with secondary antibodies (Li-Cor Biosciences, Lincoln, NE, USA) in 1% BSA for 1 h. Proteins were visualized using the Odyssey Infrared Imager (Li-Cor Biosciences). The fluorescence was quantified using the imaging software Odyssey V3.0 from the Odyssey Infrared Imager (Li-Cor Biosciences).

### 2.11. Statistical Analysis

Results are presented as mean values ± SEM. The level for statistical significance was defined as *p* < 0.05. Analyses were carried out using Prism GraphPad Software version 6 (San Diego, CA, USA). Differences between data groups were evaluated for significance using independent *t*-tests of data, one-way or two-way ANOVA and Newman–Keuls post hoc tests for multiple comparisons.

## 3. Results

### 3.1. Delayed Endurance Exercise Training Limits Calorie Intake and Prevents Body and Tissue Weight Gain in HFD Mice

**Figure 1:**
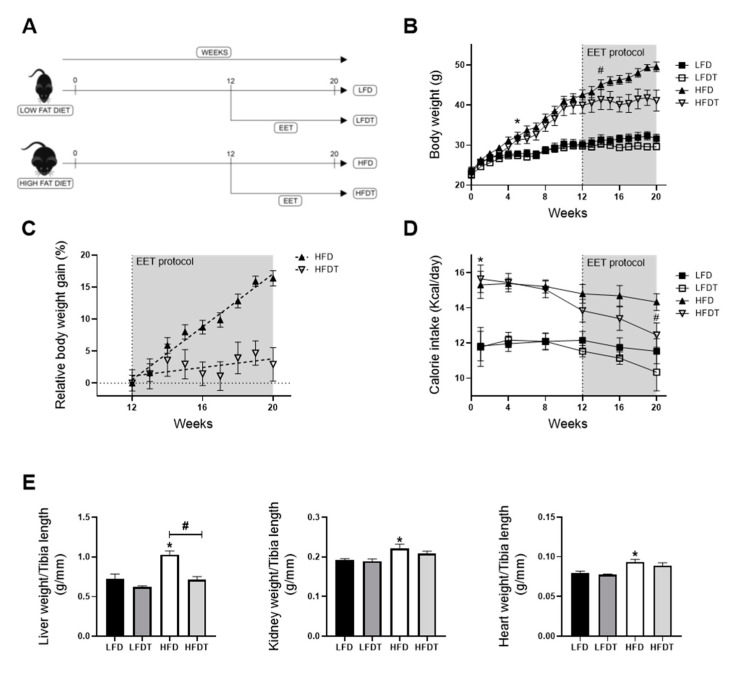
Delayed endurance exercise training (EET) prevents body weight gain and organ hypertrophy induced by HFD in mice. (**A**) Schematic representation of experimental design. Obesity in mice was induced with 12 weeks on a high-fat diet (HFD). Control mice were fed with a low-fat diet (LFD). Mice were subsequently divided in sedentary or trained groups on a treadmill for eight additional weeks (LFD, LFDT, HFD, HFDT). (**B**) Body weight evolution throughout the experimental protocol (20 weeks). Statistical analyses were performed by two-way ANOVA followed by Newman–Keuls post hoc test. (**C**) Relative body weight gain in HFD vs. HFDT mice during EET protocol normalized with body weight at week 12. The slope was determined with simple linear regression analysis. *n* = 10 in each group. (**D**) Calorie intake evolution throughout the experimental protocol. Number of calories consumed per day calculated for each mouse at different time-point (at week 1, 4, 8, 12, 16 and 20 of the experimental protocol). Statistical analyses were performed by two-way ANOVA followed by Newman–Keuls post hoc test. (**E**) Organ weights at week 20. Statistical analyses were performed by one-way ANOVA followed by Newman–Keuls post hoc test. Data are presented as means ± SEM. * *p* ≤ 0.05 versus LFD ^#^ *p* ≤ 0.05 versus HFD. *n* = 10 in each group.

The body weight and the calorie intake along the protocol, as well as tissue weights at week 20, were measured in mice fed an LFD or a HFD. The associated effects of a delayed EET treatment on these parameters were also determined. As observed in Figure 1B, a significant increase in body weight was observed from week five in HFD groups compared to LFD groups. This increase was maintained throughout the experimental protocol in HFD-fed mice compared with LFD-fed mice. This increase was observed along with a higher calorie intake in HFD mice that was significantly increased since the first week of the protocol, compared to LFD (Figure 1D). After the beginning of the EET protocol (week 12), HFDT mice showed a stabilization of the body weight. The BW of HFDT mice was significantly lower as soon as two weeks of EET compared to untrained HFD mice (Figure 1B). Figure 1C presents the percentage of body weight gain in HFD and HFDT from the beginning of EET. The linear regression analysis demonstrated that the slope of HFD was highly significant (*p* < 0.0001) but not in HFDT (*p* = 0.2397) compared with zero. These results were associated with a decrease in calorie intake at week 20 in HFDT mice compared to HFD (Figure 1D). Figure 1E presents the changes in kidney, liver and heart weights of mice fed a LFD or an HFD with or without EET at the end of the protocol. As shown, the weights of kidneys and heart were higher in mice fed a HFD compared to mice fed a LFD but not in HFDT mice. Interestingly, the important hypertrophy of the liver observed in mice fed a HFD was significantly prevented by the EET.

### 3.2. Delayed EET Improves Obesity-Related Metabolic Disorders in HFD Mice

**Figure 2:**
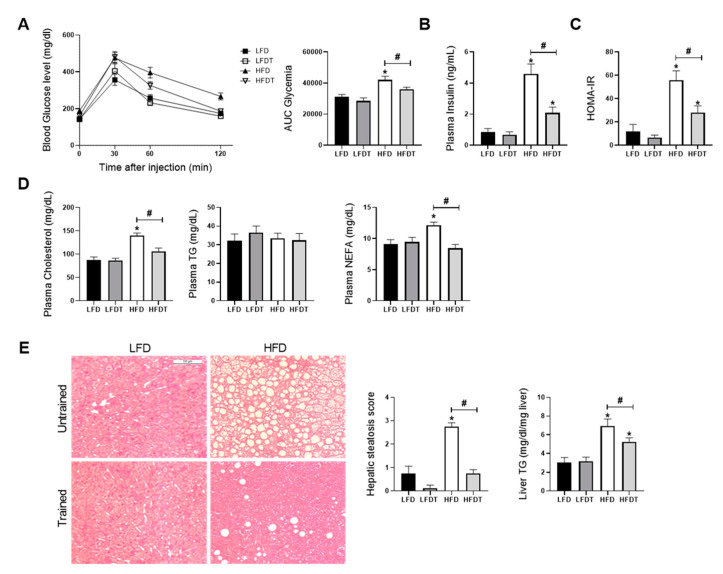
Delayed EET improves obesity-induced metabolic disorders and hepatic steatosis in HFD mice. (**A**) Glucose tolerance test at week 20. Fasted mice were submitted to an intraperitoneal injection of glucose (2 g/kg b.w.). Glycemia was measured before (0) and 30, 60 and 120 min after injection. Histogram represents the area under the curve (AUC) of glycemia from 0 to 120 min. (**B**) Plasma insulin level at week 20. (**C**) HOMA-IR index calculation. (**D**) Plasma levels of cholesterol, triglycerides (TG) and nonesterified fatty acids (NEFA) at week 20. (**E**) Representative photomicrograph (original magnification ×400; scale bar: 100 µm) of H&E staining illustrating hepatic steatosis from liver sections from mice on LFD, LFDT, HFD and HFDT at week 20 of the experimental protocol, steatosis scoring by semiquantitative analyses of lipid accumulation and quantitative analysis of triglycerides (TG) in the liver tissue. Statistical analyses were performed by one-way ANOVA followed by Newman-Keuls post hoc test. Data are presented as means ± SEM. * *p* ≤ 0.05 versus LFD ^#^ *p* ≤ 0.05 versus HFD. *n* = 10 in each group.

HFD feeding in mice is known to induce glucose intolerance, insulin resistance, dyslipidemia and hepatic steatosis. We further characterized the effects of EET on obesity-related disorders in HFD mice. To determine the impact of delayed EET on glucose tolerance, glucose tolerance tests (GTTs) were performed at week 0, before EET (12 weeks) (Figure S2) and at the end of the experimental protocol (20 weeks) (Figure 2A). At week 20, the AUC for HFD fed mice was significantly higher than for LFD groups. However, an improvement of glucose tolerance by EET was demonstrated, as observed by the significant decrease of AUC in HFDT mice compared to the HFD mice (Figure 2A). Consistently, these changes were associated with a significantly higher level of plasma insulin in HFD mice that was decreased by EET in HFDT (Figure 2B). Insulin resistance in HFD mice was confirmed by the calculation of the HOMA-IR (Homeostatic Model Assessment for Insulin Resistance) index (Figure 2C). Serum levels of adiponectin also correlates with insulin sensitivity in obesity and diabetes [33]. Despite this we did not find any changes in the total plasma adiponectin levels in the experimental groups. We evaluated the S_A_ index (defined as the HMW/(HMW  +  LMW) ratio of adiponectin multimers in plasma) that was reported as a more relevant indicator of insulin sensitivity [34]. The results demonstrated a reduction of the S_A_ index in HFD mice (Figure S3) indicating decreased HMW forms in favour of the LMW multimers as demonstrated by Pierard et al., 2016 [29]. This change was particularly reversed following exercise in HFD mice. The plasma levels of cholesterol, triglycerides and NEFA were also measured after EET treatment (Figure 2D). As illustrated, HFD induced a significant elevation of plasma NEFA and cholesterol levels, whereas these increases were counteracted by EET in HFDT mice. Interestingly, there was no change in TG level. Dyslipidaemia commonly leads to hepatic steatosis in HFD mice and is associated with metabolic syndrome consequences. Thus, the liver steatosis score and TG content within the liver tissue were evaluated as markers of nonadipose tissue ectopic lipid accumulation. As illustrated in Figure 2E, histopathologic analyses of liver from HFD mice showed the presence of macrovesicular steatosis (large fat droplets) compared with the LFD groups. This was correlated with the steatosis score evaluation (Figure 2E). In contrast, hepatic steatosis was drastically reduced by EET in HFDT mice. Similarly, hepatic TG accumulation in HFD mice was prevented by EET. The results indicate that a delayed EET protocol applied on obese mice induces a substantial improvement of the metabolic syndrome features and obesity-related disorders. Lastly, we recorded blood pressure during the last week of the protocol. We measured the systolic, diastolic and mean blood pressure. As shown in Table S2, no significant difference was observed in the different experimental groups for each of these measurements.

### 3.3. Delayed EET Improves Obesity-Related Glomerulopathy and Renal Function

**Figure 3:**
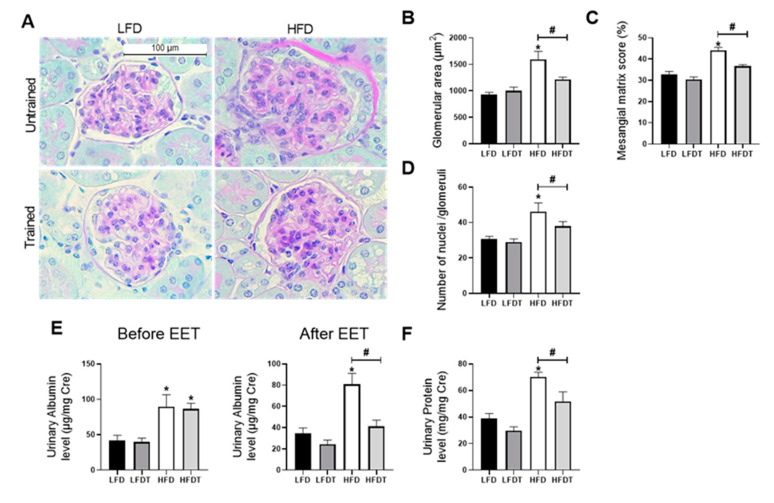
Effects of a delayed EET on renal function and glomerular histology in mice on LFD or HFD. (**A**) Representative photomicrographs (original magnification ×400; scale bar: 100 µm) of Periodic Acid Schiff (PAS) staining illustrating glomerular structure from renal cortex sections from mice on LFD, LFDT, HFD and HFDT. (**B**) Glomerular area. The glomerular tuft area was averaged from 15 glomeruli per kidney section, using one kidney section per animal. (**C**) Mesangial matrix area percentage of total glomerular area. (**D**) Nuclei count. (**E**) Quantitative measurement of urinary albumin to creatinine ratio (UACR) at week 12 (before EET protocol) and at week 20 (after EET protocol) from mice on LFD, LFDT, HFD and HFDT. (**F**) Quantitative analysis of urinary total protein to creatinine ratio at week 20. Statistical analyses were performed by one-way ANOVA followed by Newman–Keuls post hoc test. * *p* ≤ 0.05 versus LFD ^#^ *p* ≤ 0.05 versus HFD. Data are presented as means ± SEM. *n* = 10 in each group.

In order to characterize the effects of delayed EET protocol on the pathogenesis of obesity-induced chronic kidney disease, we first evaluated glomerular impairments and renal function. The morphological analysis of glomeruli revealed that mice fed a HFD without EET developed a significant increase of glomerular area along with a dense Periodic-Acid-Schiff (PAS)-positive matrix in the mesangium as well as an increase in nuclei number (Figure 3A–D). This glomerular expansion was prevented by EET in HFD mice. To further determine the effect of a delayed EET on renal function in obese mice, urinary albumin to creatinine ratio (UACR) and total proteinuria were investigated (Figure 3E). After 12 weeks on diet (before EET), both groups of HFD-fed mice presented a significant higher UACR compared to the groups of LFD-fed mice. In contrast, at week 20 (after EET intervention), the UACR in HFDT mice was significantly lower than in HFD mice, demonstrating a decreased albuminuria. The same patterns were found for urinary protein level (Figure 3F). Indeed, mice fed a HFD presented a significant increase in total proteinuria while this increase was prevented by EET. These results demonstrate that the EET protocol is beneficial to counteract the progression of obesity-related glomerulopathy and associated renal dysfunction in obese mice.

### 3.4. Delayed EET Ameliorates Renal Fibrosis, Inflammation and Oxidative Stress

**Figure 4:**
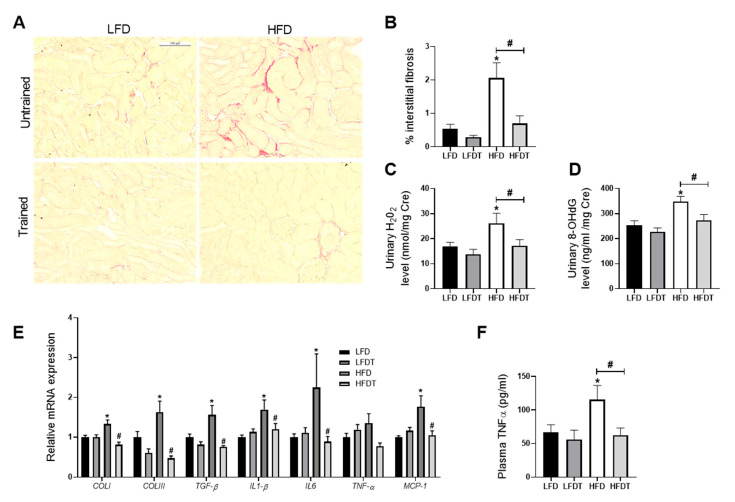
Effects of delayed EET on tubulointerstitial fibrosis, oxidative stress and inflammation from mice on LFD or HFD. (**A**) Representative photomicrographs (original magnification ×400; scale bar: 100 µm) of Sirius Red staining illustrating tubulointerstitial fibrosis in renal cortex. *n* = 10 in each group. (**B**) Quantitative analysis of the mean percentage of positive staining for Sirius red staining. *n* = 10 in each group. (**C**) Quantitative analysis of urinary H_2_O_2_ to creatinine ratio at week 20. *n* = 10 in each group. (**D**) Quantitative analysis of urinary 8-OHdG to creatinine ratio at week 20. *n* = 10 in each group. (**E**) Real-time quantitative qPCR for type I collagen (*COLI*), type III collagen (*COLIII*), transforming growth factor β (*TGFβ*), interleukin 1β *(IL-1β*), tumor necrosis factor α (*TNFα*), interleukin 6 *(IL-6*) and monocyte chemoattractant protein 1 (*MCP-1*). mRNA expressions were performed on kidney tissue from LFD, LFDT, HFD and HFDT mice normalized against 18S. *n* = 6 in each group. (**F**) TNFα plasmatic level. The TNFαconcentration was measured using indirect ELISA. *n* = 8 in each group Statistical analyses were performed by one-way ANOVA followed by Newman–Keuls post hoc test. * *p* ≤ 0.05 versus LFD ^#^ *p* ≤ 0.05 versus HFD. Data are presented as mean ± SEM. *n* = 10 in each group.

Obesity-induced chronic kidney disease is also characterized by an increased tubulo-interstitial fibrosis, inflammation and oxidative stress in HFD mice. To decipher the impact of EET on these key characteristics of the pathological progression, collagen I and III depositions were studied by morphometric analysis in Sirius red-stained renal sections as an index of the fibrotic response. As illustrated in Figure 4A, collagen accumulated in the interstitium of HFD mice. This observation was confirmed by the quantitative analysis of Sirius Red positive staining (Figure 4B). EET treatment significantly reduced collagen I and III deposition. Candidate genes expression involved in renal fibrosis (*TGF-β1*, *collagen type I* and *III*) and inflammation (*TNFα*, *IL-1β*, *IL-6* and *MCP-1*) were also measured by real-time qPCR. As observed in Figure 4E, renal mRNA levels of the profibrotic markers were all significantly decreased with EET in mice fed a HFD as well as for the pro-inflammatory markers in HFD mice. These changes were prevented by EET. The plasma TNFα concentration for systemic inflammation was also evaluated and the data reported an increased concentration of this proinflammatory cytokine in HFD mice that was significantly reduced with exercise. As markers of oxidative stress, the urinary hydrogen peroxide and 8-hydroxy-2’-deoxyguanosine (8-OHdG) levels were measured at week 20. These markers were both significantly higher in HFD mice compared to LFD mice (Figure 4C,D). The increase in urine hydrogen peroxide and 8-OHdG were also prevented by EET. The results demonstrate that EET reduces renal injuries by decreasing oxidative stress, fibrosis and inflammation in obese mice.

### 3.5. Delayed EET Reduces Renal Ectopic Lipid Accumulations

**Figure 5:**
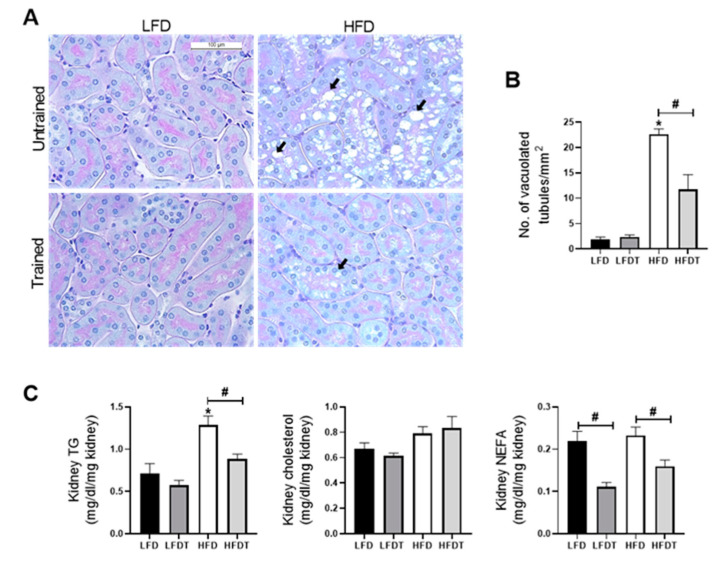
Delayed EET reduces ectopic lipid accumulation in renal tissue induced by HFD in mice. (**A**) Representative photomicrographs (original magnification ×400; scale bar: 100 µm) of PAS staining illustrating vacuolated proximal convoluted tubular cells from renal cortex sections from mice on LFD, LFDT, HFD and HFDT at week 20 (black arrow: intracellular vacuoles). (**B**) Quantitative analysis of number of vacuolated tubules per mm^2^. (**C**) Quantitative analysis of cholesterol, triglycerides (TG) and nonesterified fatty acids (NEFA) in renal tissue. Statistical analyses were performed by one-way ANOVA followed by Newman–Keuls post hoc test. * *p* ≤ 0.05 versus LFD ^#^ *p* ≤ 0.05 versus HFD. Data are presented as means ± SEM. *n* = 10 in each group.

The ectopic lipid deposition in the kidney is a key feature of obesity-induced chronic kidney disease which is associated with renal lipotoxicity. We further characterized the effect of delayed EET on tubular lipid accumulation in mice fed a LFD or HFD. As described before by Declèves et al. (2014), vacuolated tubular cells were observed in HFD groups (Figure 5A; black arrows). These tubular alterations in proximal tubules were specifically found in the renal cortex. The quantitative analysis revealed that mice fed a HFD showed a strong increase in vacuolated tubules that was significantly reduced with EET (Figure 5B). Moreover, as illustrated in Figure 5C, cholesterol, TG and NEFA renal tissue content were also measured at week 20. Data demonstrated a significant increase in TG in the HFD group that was prevented by EET. While there was no change in cholesterol content, the level of NEFA was decreased with EET in both the LFDT and the HFDT groups. Thus, EET treatment is associated with a reduced lipid accumulation in the renal tissue in obese mice.

### 3.6. Delayed EET Enhances AMPK Activity in Renal Tissue of Obese Mice

**Figure 6:**
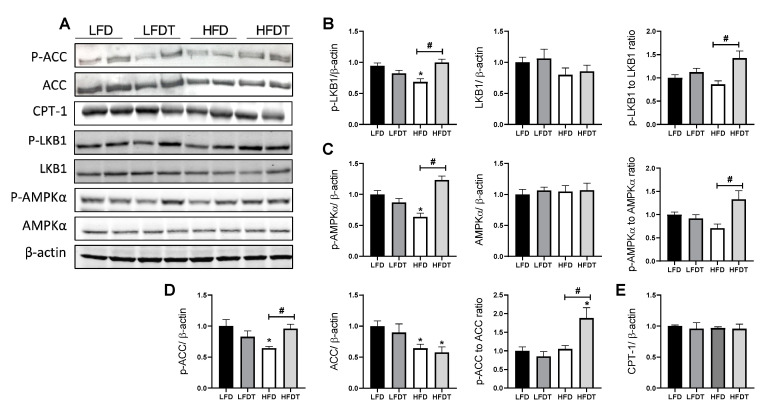
Delayed EET restores AMPK activity in the kidney of HFD mice. (**A**) Western Blot analysis of phosphor-LKB1, LKB1, phospho-AMPK, AMPK, phospho-ACC, ACC and CPT-1 in kidney tissue from mice on LFD, LFDT, HFD and HFDT at week 20. (**B**) Relative densitometry of the immunoblots representing, respectively, phospho-LKB1 protein level normalized with β-actin, LKB1 protein level normalized with β-actin and phospho-LKB1 to LKB1 ratio. (**C**) Relative densitometry of the immunoblots representing, respectively, phospho-AMPK protein level normalized with β-actin, AMPK protein level normalized with β-actin and phospho-AMPK to AMPK ratio. (**D**) Relative densitometry of the immunoblots representing, respectively, phospho-ACC protein level normalized with β-actin, ACC protein level normalized with β-actin and phospho-ACC to ACC ratio. (**E**) Relative densitometry of the immunoblots representing CPT-1 protein level normalized with β-actin. Statistical analyses were performed by one-way ANOVA followed by Newman–Keuls post hoc test. Data are presented as means ± SEM. * *p* ≤ 0.05 versus LFD ^#^ *p* ≤ 0.05 versus HFD. *n* = 6–8 in each group.

AMPK pathway dysregulation plays an important role in obesity-associated renal cell dysfunction. To better characterize the effect of a delayed EET on AMPK activity in obese mice, phosphorylation of AMPK and its main downstream target, acetyl-CoA carboxylase (ACC) as well as the main AMPK upstream kinase, the liver kinase B1 (LKB1), were measured by Western blot (Figure 6). As illustrated in Figure 6B, *p*-LKB1 protein level was significantly decreased in HFD mice compared to the control that was prevented by exercise. Moreover, total LKB1 protein level did not change between experimental groups while the *p*-LKB1 to LKB1 ratio was increased in HFDT mice compared to sedentary HFD. Mice fed a HFD exhibited a significant decrease in *p*-AMPK protein level, whereas EET treatment prevented this reduction. Total AMPK protein level was similar in each group while AMPK activity (*p*-AMPK to AMPK ratio) was increased in HFDT mice (Figure 6C). Since AMPK inhibits fatty acid synthesis and promotes fatty acid oxidation by phosphorylation of ACC, *p*-ACC and ACC protein level were also determined (Figure 6D). Consistently, protein level of phospho-ACC was reduced along with a decrease of AMPK activity in HFD group and was significantly increased with EET. Interestingly, total ACC protein level was decreased in both HFD and HFDT groups, participating in the significant increase of the *p*-ACC to ACC ratio in HFDT compared to LFD. We also investigated the protein level of CPT-1 but its expression was not regulated by HFD or EET (Figure 6E). Furthermore, the lipolysis and lipogenic markers mRNA expression were not affected by any treatment (Table S3).

### 3.7. Delayed EET Improves Autophagy Flux in Obese Mice by AMPK-Mediated ULK1 Activation

**Figure 7:**
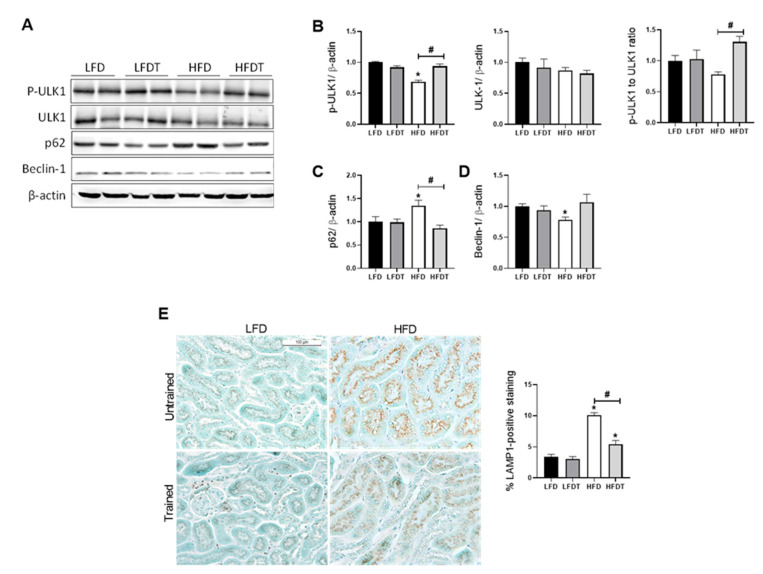
Effects of a delayed EET on autophagy and lysosomal markers in the kidney from mice on LFD or HFD. (**A**) Western Blot analysis of phospho-ULK-1 (Ser 555), ULK-1, p62 and Beclin-1 in kidney tissue from mice LFD, LFDT, HFD and HFDT. (**B**) Relative densitometry of the immunoblots representing, respectively, phosphor-ULK1 (Ser 555) protein level normalized with β-actin, ULK-1 protein level normalized with β-actin and Phospho-ULK1 (Ser 555) to ULK-1 ratio. (**C**) Relative densitometry of the immunoblots representing p62 protein level normalized with β-actin. (**D**) Relative densitometry of the immunoblots representing Beclin-1 protein level normalized with β-actin. (**E**) Representative photomicrograph (original magnification ×400; scale bar: 100 µm) showing lysosomal-associated membrane protein 1 (LAMP1)–positive staining on intracellular vacuoles and related quantitative analysis of LAMP1-positive staining. Statistical analyses were performed by one-way ANOVA followed by Newman–Keuls post hoc test. Data are presented as means ± SEM. * *p* ≤ 0.05 versus LFD ^#^ *p* ≤ 0.05 versus HFD. *n* = 6–8 in each group.

Cellular lipid accumulation and decreased AMPK activity were also associated with an impairment of the autophagy flux. Under physiological conditions, intracellular lipid storage is regulated by an autophagy process that plays a major role to prevent the deleterious effects of a lipid overload on cellular function. AMPK regulates autophagy by phosphorylating ULK-1 that mediates the initiation of autophagy. Thus, we first evaluated p-ULK1 (Ser 555) protein level in response to HFD and EET treatment. Consistently, p-ULK1 was found to be decreased in HFD mice while EET increased p-ULK1 in HFDT compared to HFD (Figure 7B). This result was corroborated with the evaluation of the autophagy marker Beclin-1, p62 for autophagy flux and LAMP-1, a specific marker of lysosomes. Indeed, Western blot analyses revealed that mice fed a HFD exhibited a significant increase in p62 protein level but not with EET (Figure 7C). p62 is a substrate of autophagy that is degraded during this cellular process. An accumulation of p62 in HFD mice revealed a stagnant autophagy process that is normalized by EET in obese mice. Beclin-1 acts during the initiation stage of autophagy by mediating the formation of a double-membrane structure that envelops cytoplasmic material to form the autophagosome. The protein level of Beclin-1 was significantly reduced in HFD and was shown to be restored by EET treatment (Figure 7D). Lastly, we demonstrated by immunohistochemistry an increased positive staining for LAMP-1 in the margins of the lipid vacuoles in proximal tubules that was significantly reduced by EET in the HFDT group (Figure 7E). These data indicate that EET enhances autophagy flux in renal tissues of obese mice possibly by AMPK-mediated phosphorylation of ULK1, ameliorating the progression of obesity-induced CKD.

## 4. Discussion

Endurance exercise training (EET) is now considered an interesting therapeutic strategy for managing obesity-related disorders. Emerging studies have demonstrated that EET is an effective strategy to prevent insulin resistance, hepatic steatosis and cardiovascular diseases [35,36]. The expected impact of exercise training treatment in obese patients is in reducing or maintaining body weight. Interestingly, EET has been shown to present beneficial effects without weight loss, suggesting independent effects of EET on obesity-related disorders [37,38]. Particularly, a redistribution of body fat along with a reduced ectopic lipid accumulation and a reduction of inflammation in tissues have been revealed with exercise [39]. Despite evidence that exercise training may improve obesity-related disorders, there is still a lack of knowledge about the underlying cellular and molecular mechanisms. Moreover, in the most experimental studies in obese rodents, animals were concomitantly exposed to EET and HFD, employing EET in a prevention setting and not as a treatment.

In contrast, here, the potential beneficial impact of EET was investigated as a therapeutic strategy on obese mice. Indeed, mice were fed a HFD for 12 weeks to achieve a significant level of metabolic and biochemical changes associated with obesity, including fat mass accumulation, glucose intolerance, insulin resistance, hepatic steatosis, ectopic lipid accumulations and associated chronic kidney disease (CKD) [15,40,41]. Thereafter, EET was applied for eight additional weeks. After 20 weeks on diets, mice exposed to a HFD presented the hallmarks of obesity-related disorders including insulin resistance, glucose intolerance and hyperlipidemia (NEFA and cholesterol). Hepatic steatosis also appeared in HFD mice as demonstrated with histological analysis and TG quantification. Several studies have already demonstrated that exercise training prevented obesity-associated disorders, including diabetes and hepatic steatosis [42,43,44]. Interestingly, in our model, delayed EET treatment in HFD mice also improved most of the metabolic changes. First, insulin resistance demonstrated in the HFD mice was reversed by EET along with improvement of glucose metabolism in HFD trained mice, as supported by HOMA-IR calculation and GTTs. The decreased insulinemia could be attributed to a lower secretion of insulin required to maintain glucose levels, suggesting a better insulin sensitivity. The exercise-induced changes in insulin sensitivity have been already extensively reported [45]. During exercise, glucose uptake is stimulated by skeletal muscle contraction. This occurs as a result of GLUT4 translocation to the muscle cell membrane [45]. Moreover, chronic exercise enhances insulin sensitivity largely due to activation of AMPK, a master regulator of glucose metabolism in the muscle [46]. The effects of exercise training on insulin resistance were also confirmed by the evaluation of adiponectin multimers distribution. As demonstrated by the S_A_ index, exercise training was associated with amelioration of the multimers distribution in favor of the HMW forms that are the most biologically active forms of adiponectin regarding glucose homeostasis [47]. EET also reduced dyslipidemia and drastically decreased intrahepatic fat content, consequently reversing HFD-related hepatic steatosis. During exercise, skeletal muscle contraction induces production of a variety of molecules called myokines that mediate beneficial responses in other organs. Experimental studies suggest that both IL-6 and Irisin might be involved in muscle/liver crosstalk mediating improvement of hepatic steatosis. In the liver, EET induces AMPK activity with enhanced mitochondrial function which lead to decreased lipogenesis and increased lipid oxidation. Hepatic AMPK activation also promotes autophagy induction via phosphorylation of ULK-1. As a result, excess lipids are eliminated by lysosomes leading to decreased ectopic lipid accumulations in the liver [48,49,50]. Furthermore, high blood pressure is another important component of metabolic syndrome, and exercise training is a recognized strategy for the prevention and treatment of hypertension [51,52]. However, in Bruder-Nascimento et al., mice fed a HFD for 24 weeks did not present any change in arterial blood pressure [53]. Similarly, we did not observe in our experimental model any significant difference of the systolic, diastolic or mean arterial blood pressure between LFD and HFD groups, neither between trained nor untrained groups, suggesting that EET has no effect on blood pressure in our experimental conditions.

Even though EET mediates improvement in key features of obesity in humans and animal models, its effects on renal function and metabolism in obesity are not well reported so far. In the general population, obesity is the second most highly prognostic factor to predict end-stage renal disease. However, the underlying mechanisms are complex and include physiological and metabolic aspects. Adiposity could directly impact the kidneys via the proinflammatory environment mediated by adipokines produced by adipose tissue, but also oxidative stress, activation of the renin-angiotensin-aldosterone system and IR [5]. These changes lead to characteristic features of obesity-induced kidney injury including the development of glomerulomegaly and ectopic lipid accumulation in the kidney, leading to renal lipotoxicity. Exercise training has been proposed to be included in renal care at any step of CKD progression [54]. A recent study has even showed that concomitant cafeteria diet exposure and EET prevented lipid depositions in the kidney in mice [55]. To further explore the adaptation of a fatty kidney to EET, we evaluated the effect of an EET protocol to an HFD-induced CKD mice model. We demonstrated that EET treatment improved key parameters of obesity-induced CKD, as evidenced by improvement of renal function as well as morphological alterations. Notably, increased proteinuria and albuminuria, which reflect both glomerular and tubular impairments, are established predictors of CKD progression [56]. Here, we demonstrated that EET reduced glomerular impairment by reducing mesangial matrix expansion and thus glomerulomegaly. At a tubular level, ectopic lipid depositions in the kidney were markedly decreased, leading to better outcomes regarding CKD progression. Consistently, we demonstrated that EET was associated with reduced interstitial fibrosis, renal inflammation and oxidative stress.

Accumulating studies have demonstrated reduction of AMPK activity in caloric excess conditions [57,58]. Activity of AMPK was reduced in kidneys of diabetic mice and humans [59]. In several studies, pharmacological AMPK activators (5-aminoimidazole-4-carboxamide ribonucleoside, Metformin, Resveratrol, Fenofibrate and AdipoRon) attenuated diabetic nephropathy and obesity-induced CKD [10,60,61,62,63]. Consistent with the previous studies, our results showed a decrease in AMPK activity in kidney tissue following a HFD as well as the decreased phosphorylation of its main target ACC and its main upstream kinase LKB1, indicating inhibition of the AMPK pathway. Interestingly, we demonstrated that EET restored the activity of AMPK in obese mice, suggesting a critical role of AMPK regulation in the beneficial effects of EET in obesity-induced CKD. AMPK plays essential roles in glucose and lipid metabolism, cell survival, growth and inflammation. AMPK also exerts a key role in mitochondria homeostasis and has been revealed to regulate autophagy in mammalian cells. Obesity induces AMPK dysregulation by multiple mechanisms independently of the AMP:ATP ratio (reviewed in [64]) including insulin resistance, inflammation, decreased adiponectin, oxidative stress and decreased activity of AMPK upstream kinase. Here, we particularly highlighted the beneficial effects of exercise training on these parameters that may reduce the detrimental intra-renal environment leading to AMPK dysregulation in favor of a proper AMPK signaling. 

Under physiological conditions, autophagy is critical for the maintenance of renal function and homeostasis [65]. Autophagy is a complex and highly regulated cellular degradation pathway, well conserved among eukaryotes, that has been intensely documented in many pathological conditions [66]. Both obesity and HFD negatively regulates autophagy in the kidney [67]. In a setting of renal lipid overload, Yamamoto et al. (2016) and our work have demonstrated the impairment of autophagy in proximal tubular cells (PTC) [10,68]. Here, we also highlighted a beneficial role of EET associated with activation of AMPK in preventing impairment of autophagy in PTC and lipid accumulation in these cells. Chronic EET was shown to induce autophagy in vivo in various tissues including muscles, liver, adipose tissue and pancreas [69]. In this study, we investigated whether EET treatment could lead to a restored autophagy flux in renal tissue in an obesity context. We demonstrated that EET regulated autophagy markers by decreasing p62 in obese trained mice and increasing Beclin-1 protein level. Activation of the autophagic flux leads to a decline in p62 level because of its degradation during the process and, contrarily, an accumulation of p62 reflects a stagnant autophagic flux [70]. Moreover, HFD also inhibits autophagy by reducing autophagosome/lysosome fusion [68]. Here, we described an increased lysosomal marker LAMP-1 that was reduced by EET in renal tissue of obese mice. Finally, we demonstrated the AMPK-mediated phosphorylation of ULK-1, an autophagy inducer, by EET in HFD mice. Thus, our data confirmed disturbance of autophagy by HFD and indicated the potential induction of autophagy process by EET via AMPK activation, leading to improvement of renal cell homeostasis. Since autophagy is a highly dynamic process, further investigations are needed to delineate the precise molecular mechanisms of exercise-induced autophagy in the kidney and the particular the role of AMPK in this process. Particularly, the use of ULK-1 knockout mice would be an interesting mechanistical strategy in order to confirm the role of AMPK-dependent autophagy in response to exercise in renal tissue. 

Finally, how skeletal muscle communication can prevent or suppress kidney injury is particularly emerging and has not been strongly investigated yet, particularly regarding muscle-kidney cross-talk during exercise. A recent work nicely demonstrated that Irisin, an exerkine, ameliorated tubule cell damage and renal fibrosis in a CKD model [71]. Interestingly, inhibition of AMPK by a specific inhibitor reduced the effects of Irisin in myocytes and hepatocytes, suggesting that Irisin could be implicated in AMPK pathway regulation [72]. However, the particular effects of Irisin on AMPK regulation in the kidney are still unexplored and needs further investigation.

## 5. Conclusions

**Figure 8:**
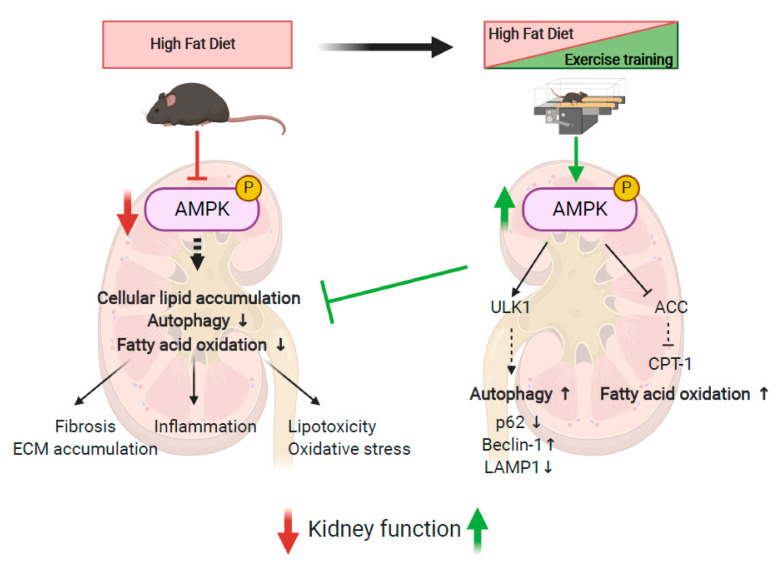
Schematical representation of the effects of HFD and EET on the underlying molecular mechanisms of obesity-induced CKD in mice. Created with Biorender.com.

Based on our data, exercise training can be considered an interesting strategy for the management of obesity-induced CKD. Kidney function was improved through reducing albuminuria, glomerular hypertrophy, inflammation, oxidative stress and fibrosis, as well as attenuating intra-renal fat content. We demonstrated that these beneficial effects implicate restored AMPK activity and autophagy induction in renal tissue (Figure 8). Exercise training may thus represent an interesting nonpharmacological alternative strategy for AMPK activation in obesity-induced CKD.

## Data Availability

Data is contained within the article or Supplementary Materials.

## Supplementary Materials

The following are available online at https://www.mdpi.com/1422-0067/22/1/350/s1, Figure S1: Effects of endurance exercise training on running velocity; Figure S2: Effects of LFD and HFD on glucose tolerance in mice; Table S1: Primer sequences for RT-qPCR analysis of mRNA expression; Table S2: Effects of delayed EET on systolic, diastolic and mean blood pressure in mice fed a LFD, LFDT or HFD and HDFT; Table S3: Effects of delayed EET on renal gene expression in mice fed a LFD, a LFDT, a HFD and a HFDT.
