# Supplementary material for: Delayed Exercise Training Improves Obesity-Induced Chronic Kidney Disease by Activating AMPK Pathway in High-Fat Diet-Fed Mice
Source: Int J Mol Sci. 2020 Dec 31;22(1):350. doi: 10.3390/ijms22010350 (PMC7795787; doi:10.3390/ijms22010350)
Supplement: Supplementary file 1 [file ijms-22-00350-s001.pdf]

Supplementary

Delayed Exercise Training Improves Obesity-Induced Chronic Kidney Disease by Activating AMPK Pathway in High-Fat Diet-Fed Mice

Florian Juszczak [1,2,*], Maud Vlassembrouck [1], Olivia Botton [2], Thomas Zwakhals [1], Morgane Decarnoncle [1], Alexandra Tassin [3], Nathalie Caron [2] and Anne-Emilie Declèves [1]

[1] Laboratory of Metabolic and Molecular Biochemistry, Faculty of Medicine and Pharmacy, Research Institute for Health Sciences and Technology, University of Mons (UMONS), 7000 Mons, Belgium; maudvlassembrouck@gmail.com (M.V.); Thomas.ZWAKHALS@umons.ac.be (T.Z.); Morgane.decarnoncle@student.umons.ac.be (M.D.); anne-emilie.decleves@umons.ac.be (A.-E.D.)

[2] Molecular Physiology Research Unit (URPhyM), Namur Research Institute for Life Sciences (NARILIS), University of Namur (UNamur), 5000 Namur, Belgium; olivia.botton@unamur.be (O.B.); nathalie.caron@unamur.be (N.C.)

[3] Laboratory of Respiratory Physiology, Pathophysiology and Rehabilitation, Faculty of Medicine and Pharmacy, Research Institute for Health Sciences and Technology, University of Mons (UMONS), 7000 Mons, Belgium; anne-emilie.decleves@umons.ac.be

* Correspondence: florian.juszczak@umons.ac.be; Tel.: +3265373580

Citation: Juszczak, F.; Vlassembrouck, M.; Botton, O.; Zwakhals, T.; Decarnoncle, M.; Tassin, A.; Caron, N.; Declèves, A.-E. Delayed exercise training improves obesity-induced chronic kidney disease by activating AMPK pathway in high-fat diet-fed mice. *Int. J. Mol. Sci.* **2020**, *22*, x. https://doi.org/10.3390/xxxxx

Received: 14 November 2020
Accepted: 26 December 2020
Published: date

Publisher's Note: MDPI stays neutral with regard to jurisdictional claims in published maps and institutional affiliations.

Copyright: © 2020 by the authors. Submitted for possible open access publication under the terms and conditions of the Creative Commons Attribution (CC BY) license (http://creativecommons.org/licenses/by/4.0/).

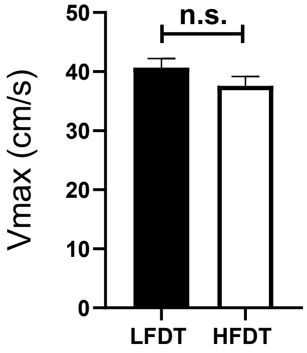

Figure S1. Effects of endurance exercise training on running velocity. Representation of maximal running velocity performance after 14 weeks on diet. *t-test*. Data are presented as means ± SEM. *ns* = non-significant. *n=10* in each group.

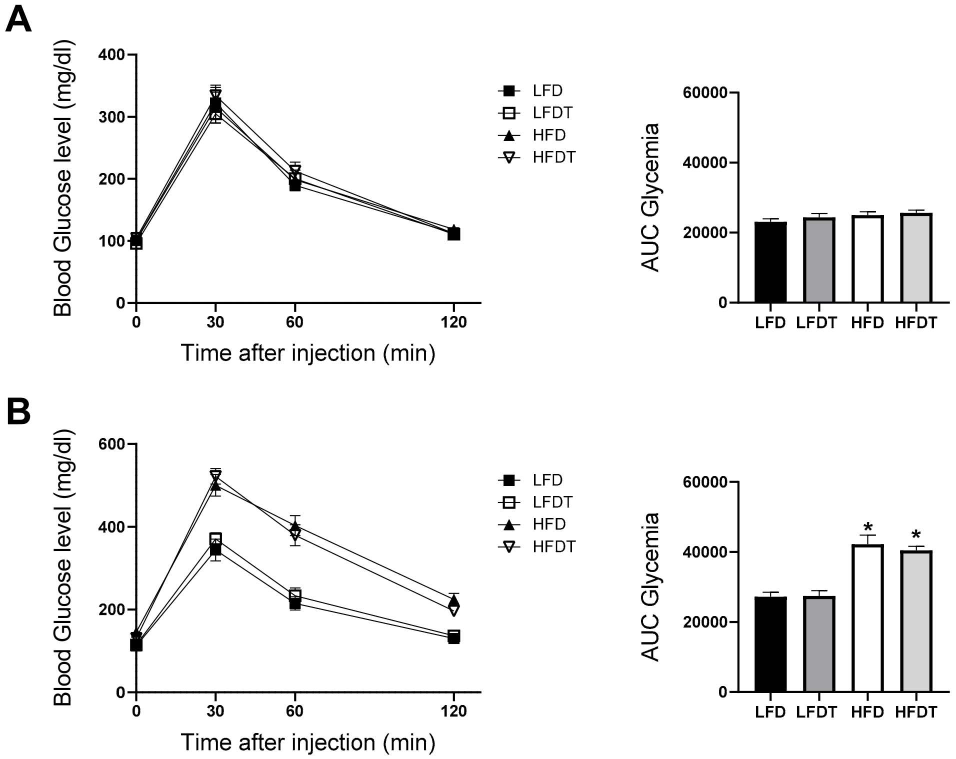

Figure S2. Effects of LFD and HFD on glucose tolerance in mice. Glucose tolerance test at week 0 (**A**) and week 12 (**B**). Fasted mice were submitted to an intraperitoneal injection of glucose (2 g/ kg b.w.). Glycemia was measured before (0) and 30, 60 and 120 min after injection. Histogram represents the area under the curve (AUC) of glycemia from 0 to 120 min

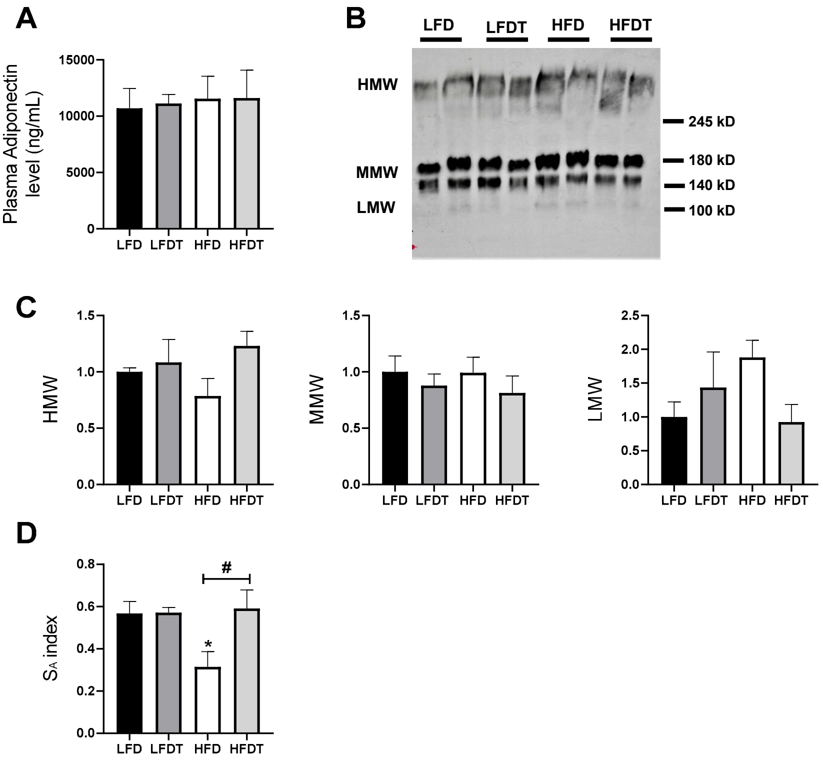

Figure S3. Total plasma adiponectin and adiponectin multimers distribution analysis at week 20. **A**. Ad plasmatic level. The total plasma Ad concentration was measured using indirect ELISA. **B**. Representative immunoblots of Adiponectin multimers expression in plasma sample. **C**. Relative densitometry of the immunoblots representing respectively HMW (High Molecular Weight), MMW (Medium Molecular Weight) and LMW (Low Molecular Weight) multimers of Adiponectin normalized with total Adiponectin protein level. **D**. S_A index was calculated as the ratio HMW/(HMW + LMW). Data are presented as means ± SEM. * $P \leq 0.05$ versus LFD $^{\#}$ $P \leq 0.05$ versus HFD. *n=6-8* in each group.

Table S1. Primer sequences for RT-qPCR analysis of mRNA expression

**Gene**		**Primer Sequences (5'-3')**
COLI	Fw	CTTGCCCCATTCATTTGTCT
	Rv	GCAGGTTCACCTACTCTGTTCT
COLIII	Fw	TGAGTCGAATTGGGGAGAAT
	Rv	TCCCCTGGAATCTGTGAATC
TGFβ	Fw	TGGAGCAACATGTGGAACTC
	Rv	GTCAGCAGCCGGTTACCA
MCP-1	Fw	CTTCTGGGCCTGCTGTTCA
	Rv	CCAGCCTACTCATTGGGATCA
IL1β	Fw	AGTTGACGGACCCCAAAAG
	Rv	AGCTGGATGCTCTCATCAGG
TNFα	Fw	TACTGAACTTCGGGGTGATTGGTCC
	Rv	CAGCCTTGTCCCTTGAAGAGAACC
IL6	Fw	GCTACCAAACTGGATATAATCAGGA
	Rv	CCAGGTAGCTATGGTACTCCAGAA
ACC (Acaca)	Fw	ATGGGCGGAATGGTCTCTTTC
	Rv	TGGGGACCTTGTCTTCATCAT
FAS (FASN)	Fw	GGAGGTGGTGATAGCCGGTAT
	Rv	TGGGTAATCCATAGAGCCCAG
CPT-1 (CPT1a)	Fw	CTCCGCCTGAGCCATGAAG
	Rv	CACCAGTGATGATGCCATTCT
18S	Fw	CGCCGCTAGAGGTGAAATTCT
	Rv	CGAACCTCCGACTTTCGTTCT

Table S2. Effects of delayed EET on systolic, diastolic and mean blood pressure in mice fed a LFD, LFDT or HFD and HDFT**.**

	LFD	LFDT	HFD	HFDT
***Systolic blood pressure** (mmHg)*	128,6 ± 10,99	138,0 ± 4,988	119,5 ± 10,86	119,3 ± 5,850
***Diastolic blood pressure** (mmHg)*	106,8 ± 10,60	117,6 ± 2,584	96,33 ± 8,750	101,7 ± 6,035
***Mean blood pressure** (mmHg)*	117,7 ± 10,78	127,8 ± 3,763	107,9 ± 9,768	110,5 ± 5,570

Measurement were performed during the last week of the experimental protocol (week 20). Systemic, diastolic and mean blood pressures were measured using a non-invasive CODA tail-cuff blood pressure occlusion system (Kent Scientific, Torrington, USA). During the week 20, measurements were taken for each animal that were acclimatized for a 1-hour period before experiments into restraining chambers. The animals were placed onto a preheated pad maintained at 30°C in a designed quiet area and blood pressure measurements were initiated when tail temperature reached 30°C (measured using an infrared sensor) and recorded at least 5 times. Mice were acclimated for at least 3 consecutive days before baseline blood pressure measurements. No statistical difference was found by One-way ANOVA analysis. *n=5* in each group.

Table S3. Effects of delayed EET on renal gene expression in mice fed a LFD, a LFDT, a HFD and a HFDT.

	LFD	LFDT	HFD	HFDT
Lipid metabolism markers				
ACC	1,000 ± 0,0918	0,9636 ± 0,1356	1,067 ± 0,1404	1,028 ± 0,0796
FAS	1,000 ± 0,0265	1,023 ± 0,1480	1,218 ± 0,2496	1,112 ± 0,1884
CPT-1	1,000 ± 0,0326	0,9017 ± 0,1308	0,8483 ± 0,1201	1,222 ± 0,2337

Real-time quantitative qPCR for Acetyl-CoA carboxylase (*ACC*), Fatty acid synthase (*FAS*) and Carnitine palmitoyltransferase I (*CPT1*). mRNA expressions were performed on kidney tissue from LFD, LFDT, HFD and HFDT mice normalized against 18S. Statistical analyses were performed by one-way ANOVA followed by Newman–Keuls post hoc test. Data are presented as means ± SEM. * P ≤ 0.05 versus LFD # P ≤ 0.05 versus HFD. *n=6* in each group.
